# Changes in Gait Variables in Patients with Intermittent Claudication

**DOI:** 10.1155/2019/7276865

**Published:** 2019-05-27

**Authors:** Bogdan Pietraszewski, Marek Woźniewski, Ryszard Jasiński, Artur Struzik, Andrzej Szuba

**Affiliations:** ^1^Department of Biomechanics, University School of Physical Education, Wrocław, 51-684, Poland; ^2^Department of Physiotherapy and Occupational Therapy in Conservative and Interventional Medicine, University School of Physical Education, Wrocław, 51-612, Poland; ^3^Department of Team Sport Games, University School of Physical Education, Wrocław, 51-684, Poland; ^4^WROVASC – An Integrated Cardiovascular Centre, Specialist District Hospital in Wrocław, Centre for Research and Development, Wrocław, 51-124, Poland; ^5^Department of Internal Medicine, Fourth Military Clinical Hospital with a Polyclinic in Wrocław, 50-981, Poland

## Abstract

**Objective:**

Intermittent claudication (IC) is a pathological symptom with a particular effect on human gait patterns. Therefore, analyzing these patterns can facilitate rehabilitation or treatment through comparison of the values of kinematic and kinetic variables of patients with the normal values of healthy people. Therefore, the aim of this study was to find differences in the values of gait variables between patients with IC and healthy people.

**Methods:**

The study included 98 patients diagnosed with peripheral arterial disease with IC. The patients traveled a distance of 6 m at a voluntary gait velocity. Ground reaction forces while the foot contacted the ground and kinematic variables of lower limb movements were recorded. The values of normal gait variables were computed based on the results obtained in a group of 30 healthy people.

**Results:**

Patients used a gait velocity below the norm for healthy people. The velocity during the lower limb swing and the step and stride length in patients with IC were below the norm. Differences were also found in the ranges of motion between patients with IC and healthy people for the pelvic obliquity, pelvic rotation, hip flexion-extension, hip abduction-adduction, hip internal-external rotation, knee flexion-extension, ankle dorsi-plantar flexion, and foot progression angles.

**Conclusions:**

The presented kinematic and kinetic characteristics measured by gait variables suggest differences between patients with IC and healthy people. Considering kinematic and kinetic gait variables during the rehabilitation process would facilitate the development of a more economic gait technique (with increased stride length and range of motion in the lower limb joints) to obtain the desired rehabilitation effects. Patients with IC should receive rehabilitation oriented towards improving mobility and increasing muscle strength in selected lower limb joints to increase gait velocity and stride length.

## 1. Introduction

As a basic form of human locomotion, gait is an efficient method of body movement. Gait velocity is a mathematical product of mean step length and step frequency. Energy cost for a gait activity would be minimized when self-selected or preferred cadences are used. Human gait is characterized by relatively low vertical movement velocities. Displacement of the center of mass of the human body occurs by means of cyclic movements of the lower limbs marked by repeated accelerations and decelerations. A cyclic movement of human body segments with respect to specific joints can be recorded by cinematographic methods under laboratory conditions [1]. Therefore, analysis of human gait movement patterns can be helpful in the process of rehabilitation or treatment through comparison of the values of kinematic and kinetic variables of patients with the norms for healthy people. Some gait variables can show interindividual differences depending on age, sex, diseases, or injuries [2].

Intermittent claudication (IC) is a pathological symptom that has a particular effect on human gait patterns [3]. IC is a common symptom of peripheral arterial disease (PAD) and is characterized by limb pain that is elicited by exercise and resolves after rest. However, unspecific leg symptoms are also common. Patients are often unable to continue their gait without frequent stopping. PAD is a relatively common disorder, in which insufficient blood flow to legs is caused by obstruction or narrowing of the arteries. Atherosclerosis is the main cause of PAD, and patients with PAD frequently suffer from other cardiovascular diseases, including ischemic heart disease and cerebrovascular disease [4].

IC is caused by exercise-induced ischemia of the lower limb muscles. Impaired blood flow and decreased oxygen supply to working muscles cause pain in ischemic muscles and hamper the patient's ability to walk [5]. Patients with IC demonstrate limited walking distance, lower physical activity levels [6, 7], muscle weakness [8–10], impaired balance [11], reduced quality of life [12], and an increased risk of death [13]. It is interesting, however, that limb blood flow parameters do not correlate with walking distance [14]. It is possible that other factors, such as abnormal gait patterns, are also responsible for walking impairment.

PAD with IC can elicit further gait adaptations including slower walking velocity, shorter step length [15–17], reduced calf muscle ability [6, 10, 11, 18, 19], reduced ability to swing the lower limbs forward [19], and decreased hip extension [6]. These gait adaptations are present even in the absence of pain but worsen as IC pain increases [10, 11, 17, 19, 20]. Patients with IC also present lower isokinetic peak torque levels during knee flexion, dorsiflexion, and plantar flexion than control patients [21]. Câmara et al. [21] and Dziubek et al. [22] suggest recommending that patients with IC increase the muscle strength of their lower limbs.

The main objective of the rehabilitation of patients with IC is an increase in the claudication distance (CD) and maximum walking distance (MWD). Paying attention to kinematic and kinetic gait variables during rehabilitation can help patients develop a more economic movement technique. Elimination of the deficiencies in the movement technique through strengthening specific muscle groups [23] may lead to increased CD and MWD [24].

Modern measuring equipment (motion analysis systems connected with force plates) allows for the analysis of a very large number of variables (kinematic and kinetic) describing the gait of the examined person. Consequently, it is unnecessary to perform many different tests and examinations to get a full picture of the gait characteristics. Furthermore, the patient does not have to perform a significant number of cumbersome examinations. It becomes possible to observe certain gait technique abnormalities based on a simple walk over a short distance along a straight line. The focus only on selected specific aspects (e.g., lower limb power) may make it impossible to detect the causes and effects of the abnormalities, since the results are incomparable due to different measurement conditions.

Therefore, this work focuses on the gait analysis in patients with IC using a simple test of walking a short distance along a straight line. The measuring equipment will enable the recording of kinematic and kinetic variables, which will help provide a full picture of the gait of the examined person. The aim of this study was to find differences in gait variables and patterns between patients with IC and healthy people.

## 2. Materials and Methods

The study included 98 patients diagnosed with PAD with IC. The mean age of the study participants was 68.0 ± 8.4 years, with body height of 1.67 ± 0.08 m, body mass of 76.8 ± 13.9 kg, BMI of 27.3 ± 4.0 kg/m^2^, Ankle Brachial Index (ABI) of left side of 0.7 ± 0.19, and ABI of right side of 0.7 ± 0.19. Study participants were selected such that PAD with IC was their only disease affecting the locomotor system. The patients' task was to cover the distance of 6 m at voluntary gait velocity. The following kinematic gait variables were recorded: pelvic obliquity, pelvic tilt, pelvic rotation, hip flexion-extension, hip abduction-adduction, hip internal-external rotation, knee flexion-extension, ankle dorsi-plantar flexion, and foot progression angles. Anthropometric measurements in the area of the lower limbs provided input data for kinematic gait analysis: bi-iliocristal width, pelvis height, lower limb length, knee width, and bimalleolar width [2]. Ground reaction forces were recorded during contact of the foot with the ground.

The measurements were performed in the Biomechanical Analyses Laboratory (with PN-EN ISO 9001:2009 certification) of the University School of Physical Education in Wrocław, Poland. Before the tests, each participant was familiarized with the research goals, was informed about the purpose of the study, and had provided written permission for the tests. The study design was approved by the Senate's Research Bioethics Committee, and the procedures complied with the Declaration of Helsinki regarding human experimentation.

The kinematic gait variables of study participants were measured using a video recording methodology by means of the BTS Smart-E motion analysis system (BTS Bioengineering, Milan, Italy). The measurement system was composed of six infrared (1.1 *μ*m) digital cameras with a frame rate of 120 Hz, two Network Camera AXIS 210A (Lund, Sweden) cameras operating in the range of visible light with a frame rate of 20 Hz, and two force Kistler 9286A plates (Winterthur, Switzerland). The sampling frequency for the signal from the force plates was set at 200 Hz. All devices performed synchronous measurements of the variables. Twenty-two photoreflective markers were attached to the body of the study participant according to the Davis model [1, 2, 25]. The research stations contained software: BTS Smart Capture, data collection; Smart Tracker, marker tracking; and Smart Analyzer, analysis and data processing. Data recordings from all devices were synchronized by the central processing unit.

The gait of patients was recorded during a set of eight attempts, with 4 to 6 gait cycles. Each measurement set had to contain at least three attempts where the foot contact with the force plate was suitable for recording ground reaction forces. This ensured determination of the support and swing phases. Means and standard deviations were calculated and averaged over the gait cycles for each gait variable. Angle-time characteristics depicting the dynamic range of movement at the main subject's joints were then acquired. All graphs were averaged over cycles and expressed as percentages of the gait cycle.

Muscle torque values for hip, knee, and ankle joints during gait were computed based on the position of the axis of rotation of the joint and the vector of the net ground reaction forces.

The values of normal gait variables for slow velocity (ca. 1 m/s) used in the present study for healthy people had been determined in the Biomechanical Analyses Laboratory of the University School of Physical Education in Wrocław, Poland. The norms were determined specifically for the activities and measurements made in that laboratory and were prepared for those purpose. The values of normal gait variables were computed based on the results obtained in a group of 30 healthy people (age: 22.0 ± 1.0 years, body height: 1.79 ± 0.06 m, body mass: 77.8 ± 9.2 kg, and BMI: 24.3 ± 2.2 kg/m^2^). Some of the norms were published in previous studies [1]. The group of patients was characterized by similar mean values of body mass as the control group (the differences were not statistically significant). However, the control group was characterized by significantly higher mean values of body height (*p* < 0.0001) and lower BMI (*p* < 0.001) from the patients group. The gait velocity of the control group was selected deliberately so that it was as close as possible to the gait velocity of the patients. It has been demonstrated that gait velocity has significant impacts on the kinematic and kinetic gait patterns [1, 26, 27]. Other variables such as age only indirectly differentiate gait because their relationship with gait variables results from differences in the gait velocity of the examined person [28]. In particular, kinematic angular variables characterizing the knee and ankle joints are positively correlated with gait velocity [29, 30]. Hanlon and Anderson [31] also showed significant relationships between the range of motion of the lower limb joints and gait velocity.

The normality of the distribution of each variable was tested using the Shapiro-Wilk and Lilliefors tests. Because the data were not normally distributed, the Mann-Whitney* U* test was applied to evaluate the differences in gait variables between the patients and healthy people. Statistical significance was determined at an alpha level of 0.05.

## 3. Results

The mean values of kinematic variables (±SD) of the patients' gait with the norms for healthy people are presented in Table 1.

Statistically significant differences were recorded between the gait velocity of patients with IC and the normal values. Patients were characterized by gait velocity below the norm for healthy people. For patients with IC, the values of the velocity of the swing lower limb and step and stride length were below the norm. Differences for other kinematic variables of patient gait were not statistically significant from those of healthy people. It should be noted that, in healthy people, the values of the variables for the left and right sides of the body are virtually identical, which indicates a correct symmetrical gait pattern (Table 1).

**Figure 1:**
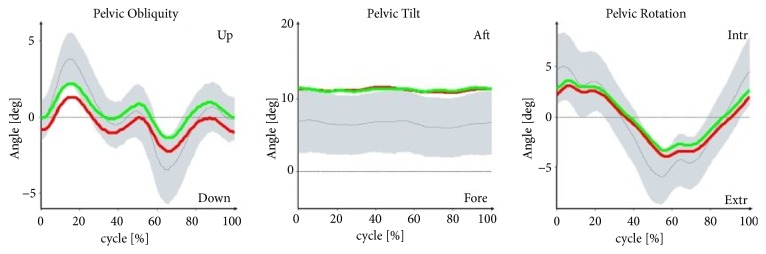
Pelvic alignment (frontal, sagittal, and transverse planes) versus gait cycle (0-65%: support phase, 65-100%: swing phase) in patients with IC compared to healthy people (norm values). The red lines are the mean values for IC patients on the right side, the green lines are the mean values for IC patients on the left side, the gray lines are the mean norm values, and the gray fields are the ±SD for the norm values.

**Figure 2:**
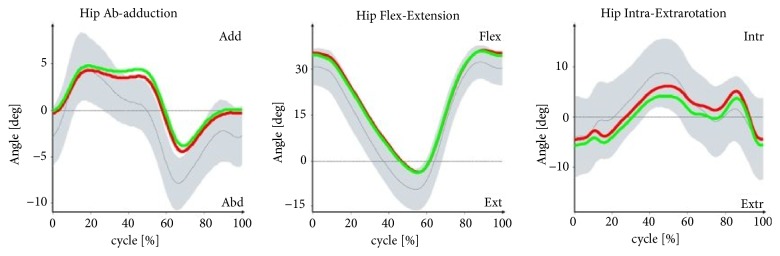
Changes in angles in hip joints (frontal, sagittal, and transverse planes) versus gait cycle (0-65%: support phase, 65-100%: swing phase) in patients with IC compared to healthy people (norm values). The red lines are the mean values for IC patients on the right side, the green lines are the mean values for IC patients on the left side, the gray lines are the mean norm values, and the gray fields are the ±SD for the norm values.

**Figure 3:**
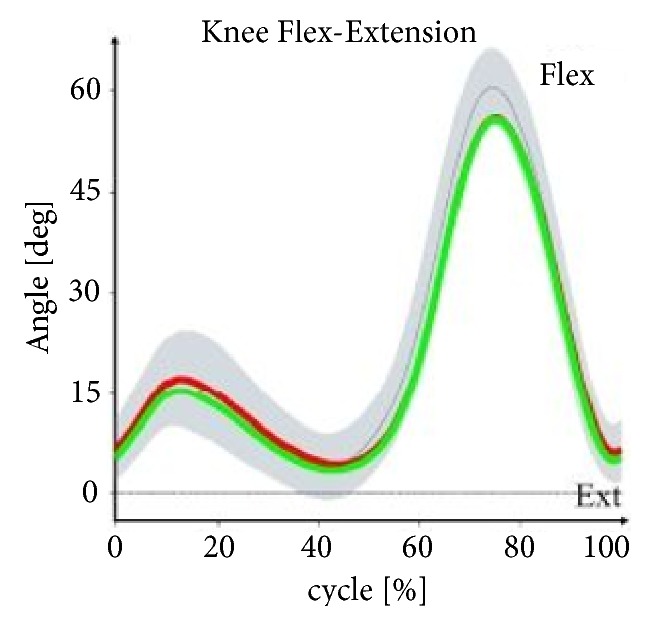
Changes in angles in knee joints (sagittal plane) versus gait cycle (0-65%: support phase, 65-100%: swing phase) in patients with IC compared to healthy people (norm values). The red lines are the mean values for IC patients on the right side, the green lines are the mean values for IC patients on the left side, the gray lines are the mean norm values, and the gray fields are the ±SD for the norm values.

**Figure 4:**
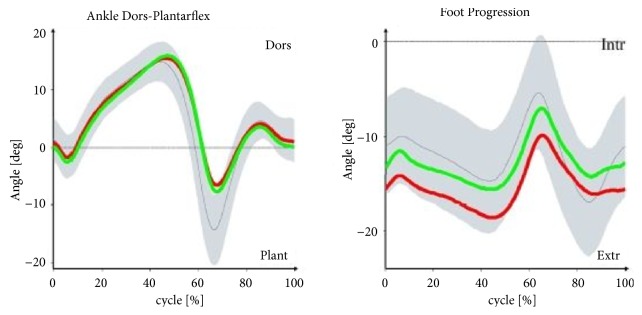
Changes in angles in ankle joints (sagittal and transverse planes) versus gait cycle (0-65%: support phase, 65-100%: swing phase) in patients with IC compared to healthy people (norm values). The red lines are the mean values for IC patients on the right side, the green lines are the mean values for IC patients on the left side, the gray lines are the mean norm values, and the gray fields are the ±SD for the norm values.

Figures 1–4 illustrate the profiles of changes in the angular values of the lower limbs in patients with IC (compared to healthy people; norm values) during an average gait cycle. Instantaneous changes in joint angles in the area of the lower limb were recorded: hip joint (in the frontal, sagittal, and transverse planes), knee joint (in the sagittal plane), and ankle joint (in the sagittal and transverse planes).

In the support phase, lowering of the pelvis is observed in the frontal plane in patients with IC. Increased adduction was found at the boundary between the support and swing phases in both the left and right hip joints. In the sagittal plane, the patients' pelvises were excessively displaced to the rear in both the support and swing phases. Limited extension occurred in the right and left hip joints at the end of the support phase. In the area of the right and left knee joint, angular patterns for the support and swing phases were normal or near normal. Excessive dorsal flexion was observed in the ankle joints in study participants at the boundary between the support and swing phases. In the transverse plane, rotation of the pelvis in patients with IC was normal, although substantial pronation was observed at the boundary between the support and swing phases. Angular patterns connected with pronation and supination for the entire gait cycle were normal. Furthermore, the right foot in the patients was excessively positioned to the outside in the support phase.

Statistically significant differences were found between the ranges of motion of patients with IC and the norms for the following movements (Table 2): pelvic obliquity (for the right and left side of the pelvis), pelvic rotation (for the right and left side of the pelvis), hip flexion-extension (for the right hip joint), hip abduction-adduction (for the left hip joint), hip internal-external rotation (for the right hip joint), knee flexion-extension (for the right and left knee joints), ankle dorsi-plantar flexion (for the right and left knee joint), and foot progression angles (for the right ankle joint). The differences for other movements in the joints of the lower limbs were not statistically significant.

**Figure 5:**
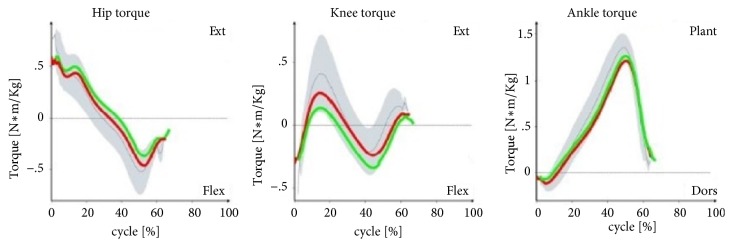
Patterns of relative values of torques in the hip, knee, and ankle joints versus gait cycle (0-65%: support phase, 65-100%: swing phase) in patients with IC compared to healthy people (norm values). The red lines are the mean values for IC patients on the right side, the green lines are the mean values for IC patients on the left side, the gray lines are the mean norm values, and the gray fields are the ±SD for the norm values.

Muscle torques developed by the flexors and extensors of the hip joints in patients with lower limb ischemia did not differ from the norms for proper gait or were at their thresholds. In the left and right knee joints, the muscle torque developed by the extensors of the left joint in the first part of the support phase was relatively low. The patterns for the torques of the plantar and dorsal flexors of both feet did not depart from the norm for the correct gait (Figure 5).

**Figure 6:**
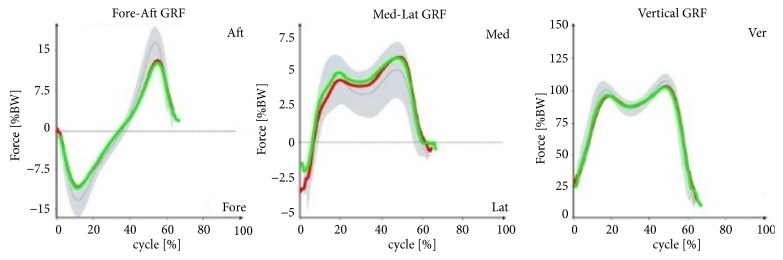
Patterns of relative values of the components of ground reaction forces versus gait cycle (0-65%: support phase, 65-100%: swing phase) in patients with IC compared to healthy people (norm values). The red lines are the mean values for IC patients on the right side, the green lines are the mean values for IC patients on the left side, the gray lines are the mean norm values, and the gray fields are the ±SD for the norm values.

At the end of the support phase, the anterior-posterior ground reaction force was lower than the norm for healthy people. Other components of ground reaction forces (vertical, anterior-posterior, and lateral) recorded during the support phases were within the norm (Figure 6).

## 4. Discussion

The gait of patients who were diagnosed with IC is characterized by a lower velocity than that for the pattern observed in healthy people. Compared to the gait norm, a noticeably lower velocity of the swinging leg and shorter step and stride lengths were also found. Other kinematic gait variables in patients with IC did not show substantial differences from the norm for healthy people (Table 1). Crowther et al. [6] and Gommans et al. [3] also demonstrated that patients with IC walked with a significantly lower velocity and shorter step length than healthy people.

With the patterns presented in Figures 1–4 concerning changes in angular values in the lower limb joints in patients with IC, the movement technique can be compared with the normal movement technique for healthy people. In most cases, patterns for patients with IC were slightly extended or were at the boundary of the norm for healthy people. An analogous tendency was observed for relative values of muscle torques in the sagittal plane (Figure 5) and the components of ground reaction forces (Figure 6). Therefore, it can be concluded that the gait of patients with IC differs from the correct pattern for healthy people. A less pronounced extension of the hip joints at the end of the support phase compared to the corresponding extension of the norm is especially worth noticing. At the end of the support phase, the anterior-posterior ground reaction force was also lower than the norm for healthy people. This may be attributable to the above-mentioned shorter step length through a reduced ability to swing the lower limbs forward.

Crowther et al. [6] noted that patients with IC showed significantly reduced displacement of ankle plantar flexion, knee range of motion, and hip extension during the gait cycle than healthy subjects. Furthermore, Gommans et al. [3] reported reductions in ankle plantar flexion, ankle range of motion, and knee range of motion in patients with IC compared to those of the control group. In this study, the ranges for the following angles were lower for patients with IC than for healthy people: pelvic obliquity (right and left side of the pelvis), pelvic rotation (right and left side of the pelvis), hip flexion-extension (right hip joint), hip abduction-adduction (left hip joint), hip internal-external rotation (right hip joint), knee flexion-extension (right and left knee joints), ankle dorsi-plantar flexion (right and left knee joints), and foot progression angles (right ankle joint). Therefore, patients with IC are characterized by limited mobility of the lower limb joints compared to that of healthy people.

The kinematic and kinetic characteristics of gait in the patients with IC demonstrated that these people should undergo rehabilitation oriented towards improving mobility and increasing muscle strength in selected lower limb joints. The patterns of kinematic and kinetic variables slightly differed from or were at the threshold of the norm. This means that patients with IC did not experience substantial problems with correct gait but did have problems with reach (distance of the route). Gommans et al. [3] did not find a significant increase in EMG amplitude for the medial gastrocnemius and tibialis anterior muscles during walking in patients with IC compared to healthy people. To date, the majority of studies on the gait of patients with IC have reported that kinematic and kinetic variables are significantly altered in patients during pain-free walking [3, 6, 10, 11, 15–19, 28, 32]. This difference compared to our study is likely to be caused by the use of different measurement methods (such as Gardner Treadmill Test, 6-Minute Walk Test, different walking distance) and devices (such as portable walkway system, isokinetic dynamometer, digital video cameras not infrared, other motion capture systems with a different number of cameras, other force plates with different sampling frequency, and different number of force plates). The present study focuses on a gait analysis in patients with IC using a simple test of walking a short distance along a straight line and modern measuring equipment that allows for the recording of kinematic and kinetic variables, revealing the full picture of the gait characteristics.

However, slight deviations for the norm in the gait technique in patients with IC can play a more important role as the distance to be covered increases. It can be presumed that a small energy expenditure that results from a less effective movement technique (which manifests mainly in the form of a lower gait velocity and step length) compared to the cyclic movement may accumulate and reach substantial values. Marconi et al. [33] estimated that the energy cost of walking is almost 40% greater in patients with IC.

The differences between kinetic and kinematic gait variables in patients with IC compared and the normal values for correct gait may provide a valuable indication for choosing a correct method to rehabilitate this group of patients. For example, the introduction of moderate-intensity walking training may lead to an increase in the distance covered by patients [34, 35]. Pharmacological treatment of IC does not have a significant effect on gait impairments [36, 37]. A number of reviews have reported the influence of exercise therapy for the treatment of patients with IC [25, 38–45]. Gommans et al. [38, 39] suggested that supervised exercise therapy led to a larger improvement in walking distance than other forms of exercise therapy. Furthermore, Dörenkamp et al. [46] stated that the benefits of supervised exercise therapy in patients with IC are underestimated. Tew and Abraham [44] concluded that efforts should be made to provide patients with access to a supervised exercise program or to promote self-managed walking when supervised exercise is not possible. However, King et al. [41] reported a lack of improvement in patients with IC gait variables after a 3-month supervised exercise program. Furthermore, Bulińska et al. [24] argued that Nordic walking training is as effective as the standard treadmill training for patients with IC.

One limitation of this study was using gait variable norms for healthy people developed in the Biomechanical Analyses Laboratory of the University School of Physical Education in Wrocław, Poland. One should realize that these values are suitable only for this laboratory and the measurement system that was used. Depending on the place and the measurement system, the mean values of norms for kinetic and kinematic variables can differ. Notably, the discussed variables are averaged patterns for the entire group of patients with IC. Much greater deviations from normal values were observed in the individual results of the patients. Therefore, one should use an individual approach for each case.

## 5. Conclusions

The presented kinematic and kinetic characteristics of gait variables in patients with IC point to differences from the norms for healthy people. Paying attention during the rehabilitation process to kinematic and kinetic gait variables would help obtain the desired rehabilitation effects through the development of a more economic gait technique. Patients with IC should receive rehabilitation oriented towards improving mobility and increasing muscle strength in selected lower limb joints to increase gait velocity and stride length.

## Figures and Tables

**Table 1:** Mean values (±SD) of kinematic variables of gait of patients with IC compared to healthy people (normal values).

Variable:	Right lower limb		Left lower limb	
	Patients	Norm	Patients	Norm
Relative variables (%)				

Support	64.7 ± 2.3	65.4 ± 1.7	64.6 ± 2.8	65.4 ± 1.7
Swing	35.3 ± 2.3	34.6 ± 1.7	35.0 ± 2.3	34.7 ± 1.7
Double stance	15.1 ± 2.5	15.4 ± 1.8	14.2 ± 2.2	15.3 ± 1.6

Temporal variables (s)				

Stride	1.22 ± 0.13	1.18 ± 0.09	1.22 ± 0.13	1.18 ± 0.09
Support	0.79 ± 0.1	0.77 ± 0.06	0.79 ± 0.1	0.77 ± 0.06
Swing	0.43 ± 0.04	0.41 ± 0.03	0.43 ± 0.04	0.41 ± 0.03

Gait variables (m)				

Stride length	1.12 ± 0.12*∗*	1.31 ± 0.13	1.12 ± 0.12*∗*	1.32 ± 0.13
Step length	0.5 ± 0.06*∗*	0.65 ± 0.16	0.51 ± 0.07*∗*	0.65 ± 0.16

Velocity variables (m/s)				

Lower limb swing	2.3 ± 0.3*∗*	2.73 ± 0.34	2.29 ± 0.31*∗*	2.73 ± 0.34
Gait velocity	0.93 ± 0.15*∗*	1.12 ± 0.15	0.93 ± 0.15*∗*	1.12 ± 0.16

*∗* indicates significant differences between patients with IC and healthy people (norm values) at *p* < 0.05.

**Table 2:** Mean values (±SD) of the lower limb joint range of motions for patients with IC compared to healthy people (normal values).

Range of motion (°):	Right lower limb			Left lower limb		
	Patients	Norm	*P*	Patients	Norm	*p*
Pelvic obliquity	5.0 ± 1.8	6.3 ± 1.8	**<0.001**	4.9 ± 1.8	6.3 ± 1.8	**<0.001**
Pelvic tilt	2.3 ± 0.9	2.3 ± 0.9	0.86	2.2 ± 0.8	2.2 ± 0.9	0.77
Pelvic rotation	8.0 ± 3.2	11.1 ± 3.8	**<0.0001**	7.9 ± 3.1	11.0 ± 3.8	**<0.0001**

Hip abduction-adduction	10.2 ± 2.7	9.1 ± 3.1	0.1	10.1 ± 2.6	12.1 ± 2.0	**<0.001**
Hip flexion-extension	41.4 ± 4.7	47.2 ± 3.8	**<0.0001**	41.0 ± 5.3	42.3 ± 4.0	0.22
Hip internal-external rotation	16.5 ± 4.1	19.2 ± 5.3	**<0.01**	15.8 ± 4.0	14.9 ± 3.6	0.28

Knee flexion-extension	53.8 ± 5.3	57.1 ± 6.3	**<0.0001**	54.4 ± 5.6	57.2 ± 6.8	**<0.0001**

Ankle dorsi-plantar flexion	23.5 ± 4.8	28.4 ± 5.6	**<0.0001**	25.2 ± 5.3	22.0 ± 3.8	**<0.001**
Foot progression	11.1 ± 3.1	13.3 ± 4.2	**<0.05**	11.3 ± 3.9	9.7 ± 3.4	0.06

## Data Availability

The data used to support the findings of this study are available from the corresponding author upon request.

## References

[1] Pietraszewski B., Winiarski S., Jaroszczuk S. (2012). Three-dimensional human gait pattern—reference data for normal men. *Acta of Bioengineering and Biomechanics*.

[2] Davis R. B., Õunpuu S., Tyburski D., Gage J. R. (1991). A gait analysis data collection and reduction technique. *Human Movement Science*.

[3] Gommans L. N. M., Smid A. T., Scheltinga M. R. M. (2016). Altered joint kinematics and increased electromyographic muscle activity during walking in patients with intermittent claudication. *Journal of Vascular Surgery*.

[4] Norgren L., Hiatt W. R., Dormandy J. A. (2007). Inter-society consensus for the management of peripheral arterial disease (TASC II). *European Journal of Vascular and Endovascular Surgery*.

[5] Baum O., Torchetti E., Malik C. (2016). Capillary ultrastructure and mitochondrial volume density in skeletal muscle in relation to reduced exercise capacity of patients with intermittent claudication. *American Journal of Physiology-Regulatory, Integrative and Comparative Physiology*.

[6] Crowther R. G., Spinks W. L., Leicht A. S., Quigley F., Golledge J. (2007). Relationship between temporal-spatial gait parameters, gait kinematics, walking performance, exercise capacity, and physical activity level in peripheral arterial disease. *Journal of Vascular Surgery*.

[7] Lauret G. J., Fokkenrood H. J. P., Bendermacher B. L., Scheltinga M. R. M., Teijink J. A. W. (2014). Physical activity monitoring in patients with intermittent claudication. *European Journal of Vascular and Endovascular Surgery*.

[8] Hedberg B., Angquist K.-A., Henriksson-Larsen K., Sjöström M. (1989). Fibre loss and distribution in skeletal muscle from patients with severe peripheral arterial insufficiency. *European Journal of Vascular and Endovascular Surgery*.

[9] Regensteiner J. G., Wolfel E. E., Brass E. P. (1993). Chronic changes in skeletal muscle histology and function in peripheral arterial disease. *Circulation*.

[10] Chen S.-J., Pipinos I. I., Johanning J. M. (2008). Bilateral claudication results in alterations in the gait biomechanics at the hip and ankle joints. *Journal of Biomechanics*.

[11] Mockford K. A., Vanicek N., Jordan A., Chetter I. C., Coughlin P. A. (2010). Kinematic adaptations to ischemic pain in claudicants during continuous walking. *Gait & Posture*.

[12] Mays R. J., Casserly I. P., Kohrt W. M. (2011). Assessment of functional status and quality of life in claudication. *Journal of Vascular Surgery*.

[13] Muluk S. C., Muluk V. S., Kelley M. E. (2001). Outcome events in patients with claudication: a 15-year study in 2777 patients. *Journal of Vascular Surgery*.

[14] Szuba A., Oka R. K., Harada R., Cooke J. P. (2006). Limb hemodynamics are not predictive of functional capacity in patients with PAD. *Vascular Medicine*.

[15] Scherer S. A., Bainbridge J. S., Hiatt W. R., Regensteiner J. G. (1998). Gait characteristics of patients with claudication. *Archives of Physical Medicine and Rehabilitation*.

[16] McCully K., Leiper C., Sanders T., Griffin E. (1999). The effects of peripheral vascular disease on gait. *The Journals of Gerontology. Series A, Biological Sciences and Medical Sciences*.

[17] Gardner A. W., Montgomery P. S., Ritti-Dias R. M., Forrester L. (2010). The effect of claudication pain on temporal and spatial gait measures during self-paced ambulation. *Vascular Medicine*.

[18] Ayzin Rosoky R., Wolosker N., Muraco-Netto B., Puech-Leão P. (2000). Ground reaction force pattern in limbs with intermittent claudication. *European Journal of Vascular and Endovascular Surgery*.

[19] Scott-Pandorf M. M., Stergiou N., Johanning J. M., Robinson L., Lynch T. G., Pipinos I. I. (2007). Peripheral arterial disease affects ground reaction forces during walking. *Journal of Vascular Surgery*.

[20] Celis R., Pipinos I. I., Scott-Pandorf M. M., Myers S. A., Stergiou N., Johanning J. M. (2009). Peripheral arterial disease affects kinematics during walking. *Journal of Vascular Surgery*.

[21] Câmara L. C., Ritti-Dias R. M., Menêses A. L. (2012). Isokinetic strength and endurance in proximal and distal muscles in patients with peripheral artery disease. *Annals of Vascular Surgery*.

[22] Dziubek W., Bulińska K., Stefańska M. (2015). Peripheral arterial disease decreases muscle torque and functional walking capacity in elderly. *Maturitas*.

[23] Struzik A., Siemieński A., Bober T., Pietraszewski B. (2018). Ratios of torques of antagonist muscle groups in female soccer players. *Acta of Bioengineering and Biomechanics*.

[24] Bulińska K., Kropielnicka K., Jasiński T. (2016). Nordic pole walking improves walking capacity in patients with intermittent claudication: a randomized controlled trial. *Disability and Rehabilitation*.

[25] Winiarski S., Pietraszewska J., Pietraszewski B. (2019). Three-dimensional human gait pattern: reference data for young, active women walking with low, preferred, and high speeds. *BioMed Research International*.

[26] Jordan K., Challis J. H., Newell K. M. (2007). Walking speed influences on gait cycle variability. *Gait & Posture*.

[27] Kwon J. W., Son S. M., Lee N. K. (2015). Changes of kinematic parameters of lower extremities with gait speed: a 3D motion analysis study. *Journal of Physical Therapy Science*.

[28] Myers S. A., Applequist B. C., Huisinga J. M., Pipinos I. I., Johanning J. M. (2016). Gait kinematics and kinetics are affected more by peripheral arterial disease than by age. *Journal of Rehabilitation Research and Development *.

[29] Bovi G., Rabuffetti M., Mazzoleni P., Ferrarin M. (2011). A multiple-task gait analysis approach: kinematic, kinetic and EMG reference data for healthy young and adult subjects. *Gait & Posture*.

[30] Lelas J. L., Merriman G. J., Riley P. O., Kerrigan D. C. (2003). Predicting peak kinematic and kinetic parameters from gait speed. *Gait & Posture*.

[31] Hanlon M., Anderson R. (2006). Prediction methods to account for the effect of gait speed on lower limb angular kinematics. *Gait & Posture*.

[32] Gardner A. W., Forrester L., Smith G. V. (2001). Altered gait profile in subjects with peripheral arterial disease. *Vascular Medicine*.

[33] Marconi C., Ferretti G., Anchisi S. (2003). Energetics of walking in patients with peripheral arterial disease: A proposed functional evaluation protocol. *Clinical Science*.

[34] Abaraogu U. O., Dall P. M., Seenan C. A. (2016). Patient education interventions to improve physical activity in patients with intermittent claudication: a protocol for a systematic mixed-studies review. *BMJ Open*.

[35] Gommans L. N. M., Hageman D., Jansen I. (2016). Minimal correlation between physical exercise capacity and daily activity in patients with intermittent claudication. *Journal of Vascular Surgery*.

[36] Huisinga J. M., Pipinos I. I., Stergiou N., Johanning J. M. (2010). Treatment with pharmacological agents in peripheral arterial disease patients does not result in biomechanical gait changes. *Journal of Applied Biomechanics*.

[37] Yentes J. M., Huisinga J. M., Myers S. A., Pipinos I. I., Johanning J. M., Stergiou N. (2012). Pharmacological treatment of intermittent claudication does not have a significant effect on gait impairments during claudication pain. *Journal of Applied Biomechanics*.

[38] Gommans L. N. M., Saarloos R., Scheltinga M. R. M. (2014). Editor's choice - the effect of supervision on walking distance in patients with intermittent claudication: a meta-analysis. *European Journal of Vascular and Endovascular Surgery*.

[39] Gommans L. N. M., Fokkenrood H. J. P., Van Dalen H. C. W., Scheltinga M. R. M., Teijink J. A. W., Peters R. J. G. (2015). Safety of supervised exercise therapy in patients with intermittent claudication. *Journal of Vascular Surgery*.

[40] Gommans L. N. M., Scheltinga M. R. M., Van Sambeek M. R. H. M., Maas A. H. E. M., Bendermacher B. L. W., Teijink J. A. W. (2015). Gender differences following supervised exercise therapy in patients with intermittent claudication. *Journal of Vascular Surgery*.

[41] Harwood A.-E., Smith G. E., Cayton T., Broadbent E., Chetter I. C. (2016). A systematic review of the uptake and adherence rates to supervised exercise programs in patients with intermittent claudication. *Annals of Vascular Surgery*.

[42] King S., Vanicek N., Mockford K. A., Coughlin P. A. (2012). The effect of a 3-month supervised exercise programme on gait parameters of patients with peripheral arterial disease and intermittent claudication. *Clinical Biomechanics*.

[43] Parmenter B. J., Raymond J., Singh M. A. F. (2013). The effect of exercise on fitness and performance-based tests of function in intermittent claudication: a systematic review. *Sports Medicine*.

[44] Tew G. A., Abraham P. (2014). Improving functional outcomes in patients with intermittent claudication. *Journal of Clinical Outcomes Management*.

[45] Kropielnicka K., Dziubek W., Bulińska K. (2018). Influence of the physical training on muscle function and walking distance in symptomatic peripheral arterial disease in elderly. *BioMed Research International*.

[46] Dörenkamp S., Mesters E. P., Nijhuis-van der Sanden M. W. (2016). How well do randomized controlled trials reflect standard care: a comparison between scientific research data and standard care data in patients with intermittent claudication undergoing supervised exercise therapy. *PLoS ONE*.

